# Management Considerations in Nasal Bone Intraosseous Cavernous Hemangioma: A Case Report and Literature Review

**DOI:** 10.1155/carm/1774878

**Published:** 2026-01-30

**Authors:** Tae-Gyun Kim, Chang-Ho Whangbo, Mi-Kyung Ye, Seung-Heon Shin

**Affiliations:** ^1^ Department of Otolaryngology-Head and Neck Surgery, School of Medicine, Daegu Catholic University, Daegu, Republic of Korea, cu.ac.kr

## Abstract

Sinonasal intraosseous cavernous hemangioma is an uncommon vascular bone tumor with clinical, radiological, and histologic characteristics that differ from soft tissue hemangioma. This case comprises an exceptionally rare intraosseous cavernous hemangioma that develops from the nasal bone. A 66‐year‐old male patient appeared with a protruding left nasal bone region and epiphora. A complete surgical excision was accomplished through a rhinotomy incision. Histological investigation revealed an intraosseous cavernous hemangioma. The patient was pleased with the cosmetic outcome, and no recurrences were detected during the 18‐month follow‐up.

## 1. Introduction

Intraosseous cavernous hemangioma is a vascular tumor that accounts for around 1% of all benign bone tumors and usually occurs in the vertebral column and craniofacial area [1, 2]. However, hemangiomas are rather frequent benign diseases that often originate in soft tissue [3]. Hemangiomas appear in early childhood as reddish cutaneous or mucosal lesions, whereas intraosseous cavernous hemangiomas usually develop in adulthood, with a female predominance. Hemangiomas are histologically categorized into three types: capillary, cavernous, and infantile. The majority of intraosseous hemangiomas are of the cavernous type, with blood‐filled vascular chambers within the bony trabeculas [2, 4]. Intraosseous cavernous hemangiomas are asymptomatic, slow‐growing tumors that rarely require treatment unless they produce cosmetic deformity or local discomfort. Although uncommon in the head and neck region, intraosseous cavernous hemangioma frequently affects the mandible, zygoma, maxilla, and frontal bone [5]. We present a case of intraosseous cavernous hemangioma emerging from the nasal bone, resulting in epiphora due to compression of the nasolacrimal duct, as well as a review of the literature.

## 2. Case Presentation

**FIGURE 1:**
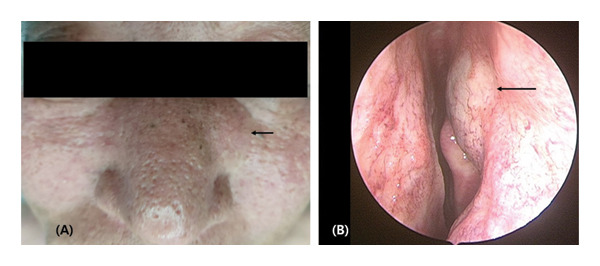
Preoperative surgical photo (A) and endoscopic finding of the left nasal cavity (B). The arrow indicates swelling of the left nasal bone (A) and lacrimal sac area (B).

A man in his 60s presented to the ophthalmology clinic with epiphora and was referred to our department for additional evaluation. He had no previous history of nasal injuries, epistaxis, facial pain, or other rhinologic symptoms, including nasal blockage, rhinorrhea, or olfactory dysfunction. He had a slowly growing, firm, fixed, nontender mass over the left nasal bone area for 20 years (Figure 1). Left epiphora first appeared around a year ago.

**FIGURE 2:**
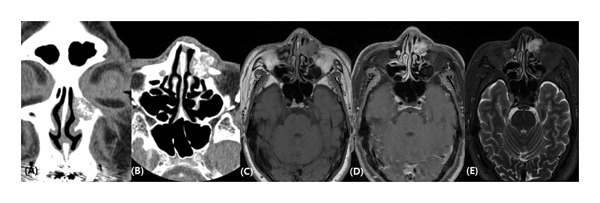
Coronal (A) and axial (B) CT showed a round honeycomb‐appearance mass in the left nasal bone compressing the nasolacrimal duct. T1‐weighted (C), contrast‐enhanced T1‐weighted (D), and T2‐weighted (E) MRI images demonstrated a heterogenous ovoid mass measuring 21 × 14 mm, protruding into the nasal cavity.

Computed tomography revealed a circular, inhomogeneous, honeycomb‐like tumor growing into the left nasal cavity. Magnetic resonance imaging detected a 21 × 14‐mm lobulated, heterogeneous enhancing mass in the left nasal bone and ethmoid bone (Figure 2). The mass spread into the surrounding subcutaneous tissue and damaged the bony wall of the nasolacrimal duct. Nasal endoscopy indicated swelling of the nasal mucosa covering the lacrimal sac (Figure 1). To rule out malignancy, an endoscopic biopsy was conducted in an outpatient setting, and the pathologist confirmed a diagnosis of cavernous hemangioma.

**FIGURE 3:**
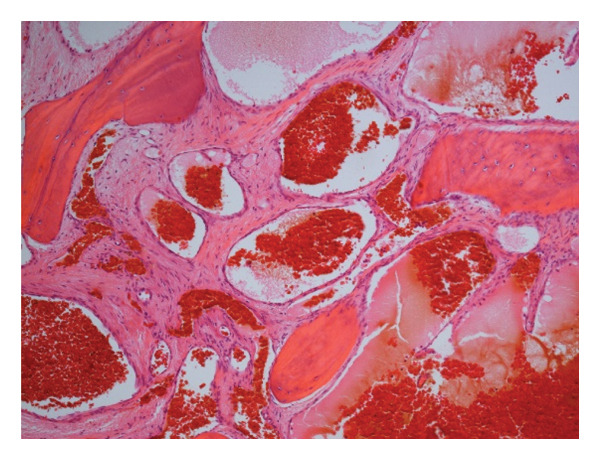
Histologic findings revealed several dilated blood vessels with a single layer of vascular space between the bony trabeculas (× 100).

Despite being benign, the patient wanted surgical excision due to the epiphora and cosmetic concerns. The mass was removed completely from the area between the nasal bone, orbit, and nasolacrimal duct using an external incision that did not injure the nasal mucosa. A well‐encapsulated mass was removed with minimal bleeding, and the site was healed without a flap or bone graft. Histological analysis revealed an expanded, dilated vascular space between bony trabeculas and fibrous stroma, with no indications of malignancy (Figure 3).

**FIGURE 4:**
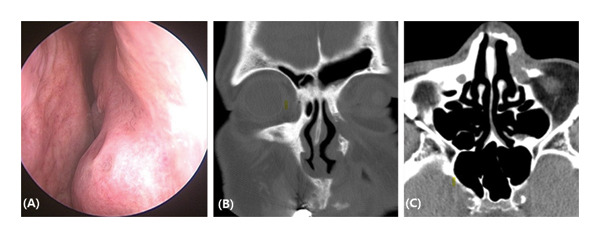
Postoperative 18‐month endoscopic finding (A), coronal (B), and axial (C) CT scans revealed no evidence of recurrence and facial deformity.

A follow‐up CT scan performed 18 months after surgery revealed no recurrence or outward facial deformities (Figure 4).

## 3. Discussion

Sinonasal intraosseous cavernous hemangiomas are extremely rare benign vascular bone tumors that can affect the nose, maxilla, or turbinates [2]. However, sinonasal hemangiomas are soft tissue vascular tumors that develop in the sinonasal mucosa. Although both are derived from vascular tissue, they have quite different clinical and pathological characteristics. Hemangiomas are frequently associated with nasal obstruction and epistaxis, whereas intraosseous cavernous hemangiomas tend to grow slowly over many years with no obvious symptoms. Hemangiomas are often reddish protruding masses that bleed easily and can be discovered with endoscopy. Intraosseous cavernous hemangiomas originate within the bone and are frequently discovered after they have reached a significant size. Hemangiomas are usually found in the nasal septum or inferior turbinate and can be easily removed with endoscopic surgery. Intraosseous cavernous hemangiomas develop within the sinonasal skeleton, such as the nasal bone or maxillary bone, and can be removed via endoscopic or external surgical techniques that require the removal of the affected bone. Complete surgical excision of both hemangiomas and intraosseous cavernous hemangiomas has a good prognosis, with low recurrence rates and few long‐term complications. Despite their characteristic CT (ground‐glass pattern) and MRI (well‐enhanced) findings, intraosseous cavernous hemangiomas should be differentiated from fibrous dysplasia, osteoma, Langerhans cell histiocytosis, and multiple myelomas [2, 3].

Since 2000, 17 cases of intraosseous cavernous hemangioma occurring in the sinonasal cavity or nasal bone, including our case, have been documented in the English literature, which is indexed in PubMed. Five cases affected the middle turbinate, five affected the inferior turbinate, six affected the nasal bone, and one affected the ethmoid sinus (Table 1). These tumors have been documented in people of various ages, from the 20s to their 70s, with no significant gender differences. Nasal obstruction was the most prevalent symptom, in 7 of the 11 cases that originated in the sinonasal cavity (63.6%). Headache and epistaxis were noted in one case each (9.1%), although two patients (18.2%) were asymptomatic and diagnosed incidentally. Two cases reported prior to 2010 were treated externally, but all other cases were effectively managed using an endoscopic technique. Although sinonasal cavity lesions have bony components, they are covered with sinonasal mucosa, making them clearly visible and accessible for complete excision via endoscopy. Among the six cases involving the nasal bone, two had epiphora, one had nasal blockage, and the other three were asymptomatic. As the tumor grows, it may compress the surrounding nasolacrimal duct, resulting in epiphora. One of these six cases was treated endoscopically, while the other five were successfully excised using an external technique. The intraosseous cavernous hemangiomas of the nasal bone are located between the skin and the nasal mucosa, making an external approach more feasible. In the case managed endoscopically, nasal mucosal surrounding the tumor was injured, necessitating attention to avoid injury to the overlying skin. External incisions have not caused any significant cosmetic concerns as compared to piecemeal removal with drills and forceps during endoscopic surgery, and the external techniques provide the benefit of en bloc removal. The surgical approach for tumor removal can be determined based on the tumor location and size, as well as patient age and sex. In our case, the intraosseous cavernous hemangioma of the nasal bone was effectively removed using an external technique with no cosmetic issues.

**TABLE 1:** Case series of sinonasal intraosseous cavernous hemangioma.

Study	Site	Age	Sex	Sx duration	Main sx	Size (cm)	Treatment
Fahmy et al. (2001) [6]	IT	25	M	2 years	Obstruction	ND	External
Kargi et al. (2005) [7]	NB	60	M	10 years	Obstruction	3 × 2	External
Stankovic et al. (2008) [8]	NB	32	F	1 year	None	2 × 3	External
Takeda et al. (2010) [9]	IT	73	F	1 month	Obstruction	4 × 5	External
Akiyama et al. (2011) [10]	MT	56	F	NA	None	3 × 3	Endoscopic
Akiner et al. (2011) [11]	IT	57	F	5 years	Obstruction	5 × 4	endoscopic
Kim et al. (2013) [12]	MT	52	M	5 years	Headache	3 × 1.5	Endoscopic
Kim et al. (2014) [13]	MT	64	M	ND	Obstruction	5 × 3	Endoscopic
Goomany et al. (2015) [14]	IT	47	F	2 years	Obstruction	4.5 × 2.0	Endoscopic
Goff et al. (2015) [15]	MT	61	M	3 years	Epistaxis	3.6 × 3.4	Endoscopic
Tomioka et al. (2020) [16]	NB	37	F	NA	None	2	Open rhinoplasty
Choi et al. (2020) [17]	NB	63	F	3 months	Epiphora	1.9 × 1.9	External
Dogan et al. (2023) [18]	NB	58	F	4 years	Swelling	1.5	Endoscopic
Caceres et al. (2023) [19]	Ethmoid	70	M	NA	Obstruction	2	Endoscopic
Bolous et al. (2023) [20]	MT	53	F	NA	None	ND	Endoscopic
Deng et al. (2025) [21]	IT	40	F	2 months	Obstruction	3	Endoscopic
Present study	NB	66	M	20 years	Epiphora	2.1 × 1.4	External

*Note:* M: male, F: female, and Sx: symptom.

Abbreviations: IT = inferior turbinate, MT = middle turbinate, NA = not applicable, NB = nasal bone, and ND = nondetermined.

Sinonasal intraosseous cavernous hemangioma is a rare vascular bone tumor. It is a slow‐growing benign expansile mass that usually requires treatment only in the presence of cosmetic concerns or specific symptoms. We report a case of intraosseous cavernous hemangioma of the nasal bone, presenting cosmetic deformity and epiphora. The lesion was completely removed via a rhinotomy incision without the need for reconstructive procedures. The patient was satisfied with the cosmetic outcome, and no recurrence was observed during the 18‐month follow‐up.

## Funding

The authors received no specific funding for this work.

## Ethics Statement

This study was approved by the Institutional Review Board and Ethics Committee of Daegu Catholic University Medical Center (CR‐25‐004).

## Consent

Written informed consent was obtained from the patient for publication of this case report and any accompanying images. A copy of the written consent is available for review by the editor of this journal.

## Conflicts of Interest

The authors declare no conflicts of interest.

## Data Availability

Data are available from the corresponding author upon reasonable request.
